# Oral Cavity Status of Type 1 Diabetic Patients Who Underwent an Oral Hygiene Tuition

**DOI:** 10.3390/healthcare10040606

**Published:** 2022-03-23

**Authors:** Bartosz Mosler, Henryk Twardawa, Agata Trzcionka, Rafał Korkosz, Mansur Rahnama, Marta Tanasiewicz

**Affiliations:** 1 Department of Conservative Dentistry with Endodontics, Faculty of Medical Sciences in Zabrze, Medical University of Silesia, Plac Akademicki 17, 41-902 Bytom, Poland; bartosz.mosler@sum.edu.pl (B.M.); htwardawa@sum.edu.pl (H.T.); rkorkosz@sum.edu.pl (R.K.); martatanasiewicz@sum.edu.pl (M.T.); 2 Chair and Department of Dental Surgery, Medical University of Lublin, Dr W. Chodźki 6, 20-930 Lublin, Poland; mansur.rahnama@umlub.pl

**Keywords:** diabetes mellitus, oral hygiene, chronic disease, dental treatment needs

## Abstract

Diabetes mellitus is a civilization disease which affects increasing number of people. Diabetes essentially influences gingival disease, periodontal disease, saliva secretion disorders and its parameters. The aim of the study is both assessing the oral cavity status of that group of patients to clearly identify their treatment needs and the effectiveness of implementation of oral hygiene training. 57 patients with type 1 diabetes and 31 healthy declared their contribution to the research. The research was conducted within two visits. The evaluation was done during clinical examination: teeth condition, oral hygiene. During the end of the first visit patients received professional oral hygiene instructions. Analysis of the clinical research in both groups showed no differences for Decayed- Missing- Filled teeth index and Dental Treatment Index. The results demonstrated disparity between the groups for the decayed teeth component and filled teeth component (symptomatically higher values in the researched group). There was statistically significant difference in the modified Sulcus Bleeding Index values analysis between both groups. Conducting hygiene instructions influenced the decline of Approximal Plaque Index and Oral Hygiene Index in the treatment group. It is essential for dentists in cooperation with diabetologists to educate patients on the necessity to maintain oral hygiene procedures.

## 1. Introduction

Diabetes mellitus is closely associated with oral health. Hyperglycaemia can cause complications related to most organ systems especially the eyes, kidneys, nerves, heart, and blood vessels [1]. High glucose levels, advanced glycation end-products, and reactive oxygen species in the periodontal tissues of patients with diabetes mellitus cause a host response that leads to inflammation associated with the development of periodontal disease [2,3]. The people with periodontal disease have a greater risk of poor blood glucose levels and diabetes-related complications among people with diabetes mellitus. There was described, a bidirectional association between diabetes mellitus and periodontal disease [3]. Several studies have also reported an association between dental caries and diabetes mellitus [4,5]. De Lima et al. [4] reported that dental caries was more prevalent among patients with diabetes mellitus than among healthy controls. Lattie et al. [5] found that the Decayed, Missing, and Filled Teeth index in patients with diabetes mellitus was twice as high as that in patients without diabetes mellitus [6]. Diabetes is classified as civilization disease, affecting more and more people all over the world. That group of diseases is a result of changes in lifestyle, nutrition habits that occurred in developing countries [7,8,9]. The increasing number of patients diagnosed with diabetes makes it necessary to educate patients about the correlation between oral cavity status and the management of general disease. Çankaya et al. observed that patients are much more aware on the effect of diabetes on oral cavity status than the reverse connection. They also pointed that dental practitioners are much more affective in providing patients with appropriate knowledge than general practitioners [10]. Increasing number of patients diagnosed with diabetes made the authors to design a study that aimed at assessing the oral cavity status of that group of patients and the effectiveness of implementation of oral hygiene training. We succeeded in conducting a clinical research that required gathering the same group of patients twice what is very difficult in epidemiological studies [11]. The oral cavity assessment was conducted with the usage of dental indices widely used in measuring dental disorders such as: Decayed- Missing- Filled Teeth Index (DMF), Dental Treatment Index (DT), Approximal Plaque Index (API), Oral Hygiene Index-Simplified (OHI-S) and modified Sulcus Bleeding Index (mSBI) [11,12,13].

## 2. Materials and Methods

### 2.1. Subject

Patients diagnosed with type 1 diabetes were included into the treatment group (TG) and healthy patients into the control group (CG). Final qualification was done on the basis of medical interview which made it possible to define the inclusion and exclusion criteria for each of the groups. 

#### 2.1.1. Treatment Group

Treatment group was composed of 57 adult patients (male and female), with diagnosed type 1 diabetes, treated with insulin therapy in following centers: Diabetic Clinic of NZOZ Med-Art in Żory, Clinic of Internal Medicine and Diabetology in Hospital no. 1 in Zabrze of Silesian Medical University in Katowice, Regional Clinic for Diabetic Patients in Zabrze, Regional Specialistic Clinics in Katowice, Specialistic Medical Centre Diasomed, Diabetic Clinic of Silesian Foundation for Children and Teenagers with diabetes in Gliwice.

##### Inclusion Criteria

Adult patients–male and female, treated with insulin therapy for at least 2 years, with compensated diabetes (assessed on the basis of glycated hemoglobin levels on the date of first dental examination), with no exacerbation stages of any chronic diseases, that given their written consent to participate in the study. 

##### Exclusion Criteria

Patient that did not agree to participate in the study, who were incapacitated, with diagnosed: diseases of salivary glands, who underwent radiotherapy of head and neck, toothless patients, people treated with medications that can cause the decrease of saliva secretion (psychotropic, anticholinergic, antihistamic and antiemic), pregnant women, patients with exacerbation of general diseases. 

##### Control Group

Control group was composed of 31 patients that were not diagnosed with diabetes. They were patients of: Medical Centre Anet-Med. In Sosnowiec and Conservative Dentistry with Endodontics Clinic of Academic Centre of Dentistry of Silesian Medical University in Bytom. 

##### Inclusion Criteria

Adult patients–male and female that were not diagnosed with type 1 diabetes, that gave their written consent to participate in the study. 

##### Exclusion Criteria

Patient that did not agree to participate in the study, who were incapacitated, with diagnosed: diseases of salivary glands, who underwent radiotherapy of head and neck, toothless patients, people treated with medications that can cause the decrease of saliva secretion (psychotropic, anticholinergic, antihistamic and antiemic), pregnant women, patients with exacerbation of general diseases. 

### 2.2. Methods

The research was composed of two stages:

1. First dental appointment during which medical interview and the questionnaire examination were done as well as the analysis of the oral cavity status of the participants. At the end of the first appointment patients from both groups were given oral hygiene instructions. The proper technique of teeth brushing was presented and each patient trained the procedure with the dentist. Patients were given all the instructions in the written form (information regarding the proper technique of teeth brushing and additional products used in oral hygiene procedures as: dental floss, interdental brushes and mouthwashes was included).
2. Second dental appointment-calculation of oral hygiene indices was carried out. It took place 6 months after the first appointment.

#### Questionnaire Examination

In that part of the study patients were asked about: sex, age, diagnosed diseases and taken medications, the course of dental treatment (frequency of appointments, causes of the dental visits), presence of blood while teeth brushing, the type of used toothbrush, usage of dental floss, mouthwashes, any other additional hygienic instruments, frequency of meals and intake of sweet liquids after evening teeth brushing. 

Following questions (in patients’ native language) were asked in that stage of examination:

1. How often do you visit a dentist? (every month/every three months/every six months/once a year/occasionally)
2. What are the reasons for your dental appointments? (toothache/problems with gingiva/check-up/other-what?)
3. Were you given any instructions regarding oral hygiene by a dentist or dental hygienist? (yes/no)
4. Did you have scaling, sandblasting and fluoridation done? If yes, when?
5. Do you observe bleeding while teeth brushing? (yes/no)
6. How often do you brush your teeth? (once a day/twice a day/three times a day/four times a day and more)
7. What kind of toothbrush do you use? (manual- soft/medium/hard; electric)
8. Do you use any additional products to maintain the proper oral hygiene? (mouthwash/interdental toothbrush/irrigator/dental floss/tongue brush’ other- what kind?)
9. Do you eat or drink (liquids other than water?) after evening teeth brushing?
10. How many meals do you eat during the day (including snacks)? (1/2/3/4/5/6/7/8)

Oral cavity assessment was done with the usage of following dental indices:

- DMFT (Decayed-Missing-Filled Teeth Index)

That index is a sum of the number of decayed, missing (because of caries) and filled teeth (permanent), where:

D-a tooth with one or more cavities (primary or secondary)
M-a tooth lost due to caries
F-a tooth with one or more fillings but with no secondary caries, a tooth with a prosthetic crown (that was applied because of caries)

- *Dental Treatment Index*

It is a measure of the success in caries treatment, where *F*–Filled, *D*–decayed.

$$Dental\;Treatment\;Index=\frac{F}{D+F}$$

Possible values of that index are 0–1. 0 means that none of the teeth with cavities was treated, while 1- all decayed teeth were filled.

- *Frequency of caries*

It is defined as the percentage of people with diagnosed caries (DMFT > 0)

$$Frequency\;of\;caries=\frac{number\;of\;teeth\;with\;diagnosed\;caries}{number\;of\;examined\;teeth}\times 100\%$$

- *API* (Approximal Plaque Index) by Lange [12]

A yes/no decision is made with regards to whether the examined interproximal surfaces is covered by plaque (+) or not (−). The index is calculated as per the following formula:

$$API=\frac{no.\;of\;plaque\;\left(+\right)sites}{no.\;of\;sites\;examined}\times 100\%$$

The examination was done using the dental probe. The minimal number of examined interproximal surfaces was 10–12, necessary between molar teeth. Quadrants 1 and 3 were examined from the oral aspect and quadrants 2 and 4 were examined from the facial aspect.

According to the results, the oral hygiene conditions of individuals could be determined as follows:

100–70%—poor oral hygiene,
69–40%—insufficient oral hygiene,
39–25%—pretty good oral hygiene,
<25%—optimum hygiene.

- OHI-S (Oral Hygiene Index-Simplified) by Green and Vermillion [12]

The index has two components, the Debris Index (DI) and the Calculus Index (CI). Each of these indices is based on numerical determinations representing the amount of debris or calculus found on the preselected tooth surface. The six surfaces examined for the OHI-S are selected from four posterior and two anterior teeth.

The examination was done using a dental mirror. Six teeth were examined, including 16 and 26 (first upper molars) on the buccal surfaces, 26 and 46 on the lingual surfaces (first lower molars), and 11 and 31 (first upper and first lower incisors) on the labial surfaces. The result of the examination was assigned a value between 0 and 3:

0- No debris or calculus,
1- Soft debris or supragingival calculus, covering not more than one third of the exposed surface,
2- Soft debris or supragingival calculus, covering not more than two thirds of the exposed tooth surface, or presence of flecks of subgingival calculus around the cervical portion of the tooth, or both,
3- Soft debris or supragingival calculus covering more than two thirds of the exposed tooth surface, or a continuous heavy band of subgingival calculus around the cervical portion of the tooth, or both.

The results obtained for particular teeth (surfaces) were added and then divided by the number of examined teeth. The possible results vary between 0 and 3 for the Debris and Calculus Indices and between 0 and 6 for the OHI-S values.

Muhlemann-Son Sulcus Bleeding Index (mSBI) was used to assess the severity of gingival bleeding. To estimate that index, a periodontal probe was passed along the gingival margin in order to provoke bleeding. The obtained results were scored with values from 0 to 3:

0- No bleeding on probing
1- Isolated bleeding spots
2- Blood formed a red line along the gingival margin
3- Heavy bleeding [11].

### 2.3. Statistical Analysis

The data analyses were performed using the R v.4.1.1 statistical computing environment. 

The significance level α for statistical tests was set at 0.05.

The Shapiro-Wilk test was used to test the normality of the distribution of variables on a continuous scale. The significance of mean differences of two normally distributed samples was estimated with the *t*-Welch test. In the case of a non-normal distribution the Mann-Whitney test was implemented in the case of independent groups and the Wilcoxon test for the dependent ones.

Data analysis was performed with the use of the following packages: psych, rstatix (for the computation of descriptive statistics, stats for the estimation of statistical tests, and ggstatsplot for graphical visualization with statistics reporting.

## 3. Results

### 3.1. Patients Characteristics and Their Hygiene Habits

The treatment group was composed of 57 patients (38 women and 19 men) diagnosed with type 1 diabetes, while the control included 31 healthy people (20 women and 11 men). Test of independence proved that both groups do not differ significantly in terms of sex.

The analysis of general diseases that patients declared to suffer from (blood hypertension, heart diseases, thyroid diseases, asthma, bleeding and clotting disorders), showed that both groups differ significantly only in frequency of occurrence of thyroid diseases (*p* = 0.0167). 

The statistical analysis proved that there were no statistically significant differences between both groups in answer for the questionnaire questions.

### 3.2. Control Group (Visit 1) vs. Treatment Group (Visit 1)

The statistically significant results of differences in means between the control group and the treatment group (two independent samples) for the first visit are presented in Table 1.

Data from Table 1 shows the significant differences between groups (*p* < 0.05) for the following variables Oral Hygiene Index (DI), Oral Hygiene Index (CI), Oral Hygiene Index (CI +DI), Modified Sulcus Bleeding Index. 

The medium number of teeth was similar in both groups (Control Group = 28, Treatment Group = 27; *p* = 0.317). No statistically significant differences were observed in Dental Treatment Index (*p* = 0.584) nor Frequency of Caries (*p* = 0.545). There were also no differences in the DMF index and its components (decayed, missing and filled teeth) between both groups of participants. On the basics of the API results it was stated that the oral hygiene in Control Group was insufficient (M = 67.80; SD = 33.60) and in Treatment Group- poor (M = 75.00; SD = 27.80) (*p* = 0.226).

### 3.3. Control Group (Visit 2) vs. Treatment Group (Visit 2)

**Figure 1:**
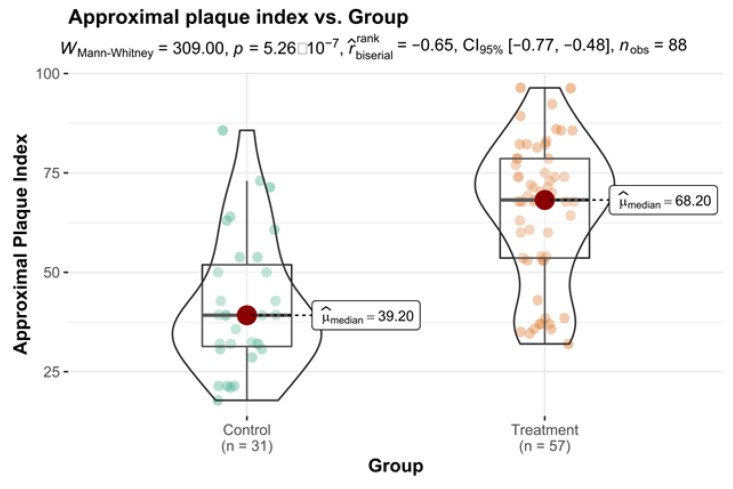
Approximal plaque index vs. Group variable with differences in means statistical reporting.

The analysis of the hygiene indices for the second appointment between the groups proved that the oral hygiene in the Control Group was pretty good (M = 39.20; SD = 20.6) while in Treatment one- insufficient (M = 68.20; SD = 20.50), and the difference was statistically significant (*p* < 0.001) (Figure 1).

Statistically significant differences were also observed in OHI-S Index values and 1 out of 2 components (CI) (Table 2).

**Figure 2:**
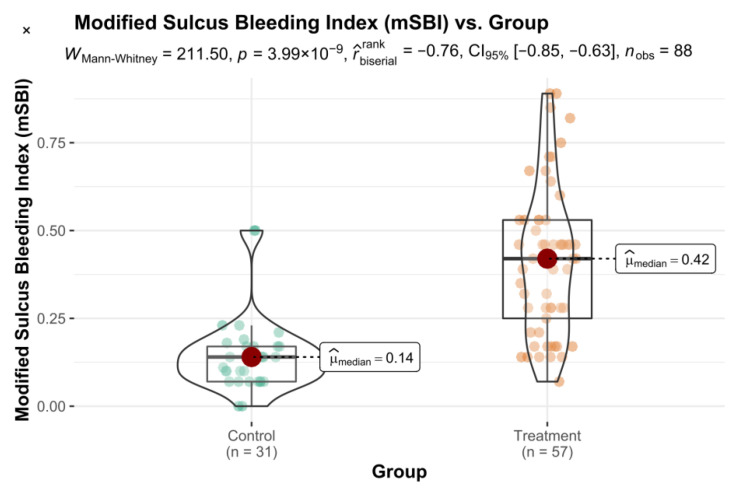
Modified Sulcus Bleeding Index vs. Group variable with differences in means statistical reporting.

The obtained results of the mSBI index were also statistically significant (Figure 2).

### 3.4. Control Group (Visit 1) vs. Control Group (Visit 2)

**Figure 3:**
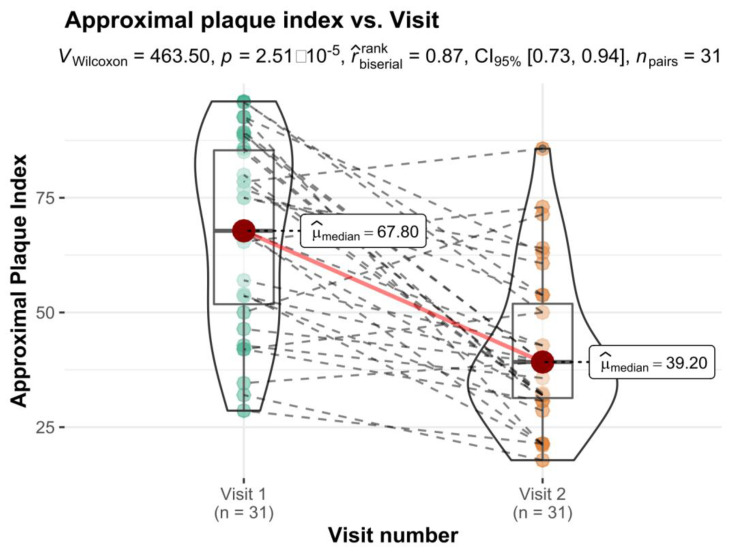
Approximal plaque index vs. Visit variable with differences in means statistical reporting.

**Figure 4:**
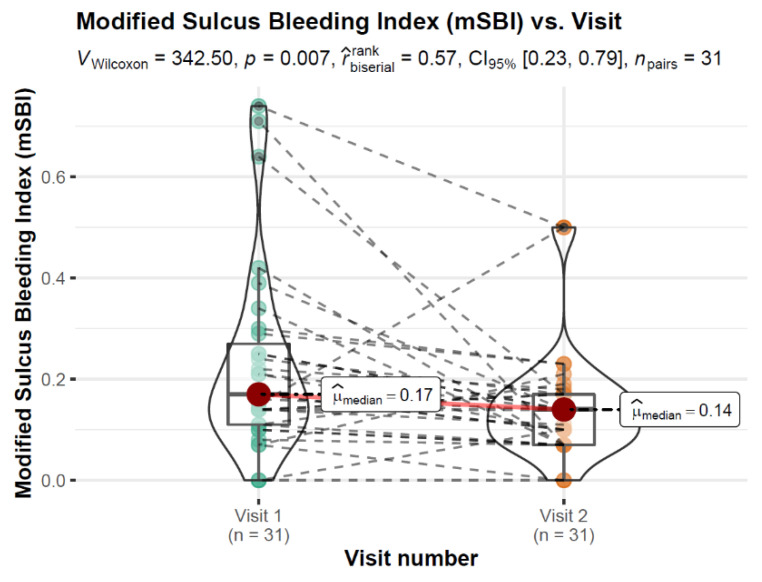
Modified Sulcus Bleeding Index vs. Visit variable with differences in means statistical reporting.

Comparison of oral hygiene indices in Control Group for 2 appointments showed statistically significant differences for Approximal Index (Figure 3) and Modified Sulcus Bleeding Index (Figure 4). No differences were observe in OHI-S (*p* = 0.801) and its components (DI: *p* = 0.159; CI: *p* = 0.408).

### 3.5. Treatment Group (Visit 1) vs. Treatment Group (Visit 2) 

There were statistically significant differences in all assessed indices in Treatment Group for the second appointment (Table 3). The improvement in API, DI, OHI-S and mSBI was observed.

## 4. Discussion

Oral hygiene status in patients diagnosed with general diseases is worse than in healthy people. Many of those diseases results in specific symptoms in oral cavity (pathologies of mucosa, periodontitis and teeth). These symptoms are very often a result of improper oral hygiene and neglect of regular visits at the dental office. It is mainly caused by the fact that the patient is completely focused on the treatment of the chronic disease [13]. 

Own results do not confirm the influence of type 1 diabetes on caries occurrence in opposite to results of de Lima et al. We did not observe differences between the groups in DMF index and its components. Similar results were obtained by Machado et al. who examined patients diagnosed with type 1 diabetes and treated with the insulin injections [14]. They did not observe any statistically significant relation between diabetes and dental caries [15,16]. Own research showed that values of caries frequency index were higher in control group: 10.70 v. 7.70. Kuźmiuk et al. who examined caries intensity and efficiency of treatment, periodontal status and oral hygiene in children and teenagers with type 1 diabetes observed statistically significant lower values of caries intensity and better periodontal status in examined group. They emphasized, that the results of studies available differ when assessing the influence of diabetes on caries frequency [17]. 

In our opinion problem of caries occurrence in diabetic patients is much more complicated and should be analyzed with regard to dietary habits of examined group. We have to remember that patients diagnosed with diabetes should follow a diet that limits or even eliminates carbohydrates that are one of the crucial factors in caries development. Having that in mind, we believe that the oral hygiene is the main factor that influences the caries occurrence in that particular group of patients. 

The analysis of oral hygiene habits of our patients proved no statistically significant differences between examined and control groups in terms of: frequency of teeth brushing, reasons for visiting the dentist, type of the using toothbrush and number of meals during the day. Researchers from Switzerland, who examined patients with type 1 diabetes from 2016–2018, also did not show differences in frequency of teeth brushing [18]. However, Kanjiarath et al., who examined 77 patients with diabetes, observed that they brushed and flossed less frequently than healthy ones [19]. Moore et al. examined 406 patients with type 1 diabetes and did not observe differences between diabetics and nondiabetics in terms of hygiene behaviors. However they reported that for patients with diabetes the cost of dental treatment was the main reason for avoiding regular chek-ups [20]. These researchers also noted that diabetic patients were unaware of the oral health complications of their disease [20]. Al Amassi et al. who examined 278 diabetic patients noticed that most of the patients were aware of the influence of diabetes on dental problems, the main source of information was the media and even though patients knew that controlling the diabetes decreased the risk of oral complication only 15.1% of them visits the dentist regularly [21]. They concluded that educational programs for diabetics (especially with a low level of education) should be implemented and dental practitioners should be more responsible for the education of the patients [21]. Similar results were obtained by Bowyer et al., who carried out a questionnaire study on 615 adults diagnosed with diabetes. They concluded that patients had poor awareness of oral care and health complications associated with diabetes. Authors claimed that there was a need of training for patients regarding the importance of good oral health [22].

Observations of the cited above authors were confirmed in our research. We observed improvement of API and OHI in patients who received oral hygiene instructions. That is why we strongly agree with the opinion that there is a need of patients education on the influence of general diseases on oral status, but also of the oral status on the treatment process of the general disease. In our opinion patients diagnosed with severe general disease are much more concentrated on its treatment and neglect other areas of their health. We observed similar behavior in hemodialized patients [13]. Our results indicate for need of creation an interdisciplinary team of doctors who would treat diabetic patients. If the patient is so concentrated on instructions received by the general practitioner (regarding the treatment of general disease) maybe it should be that group of practitioners who should inform patients of the importance of proper dental hygiene maintain and its influence on diabetes management.

Ship emphasized that dentists may play an important role in its diagnosis and treatment. Dental professionals should encourage their patients to control the level of glucose in blood, maintain proper oral hygiene, follow a diet and regularly visit a diabetologist [3]. The issue of improvement of medical care in diabetics and dental prophylaxis in diabetic patients was also discussed by Barylo et al., who emphasized that the problem is very important for the improvement of their quality of life. In their studies on diabetic patients from Ukraine they observed that those people due to the condition of the oral cavity declared the lack of self-esteem, lack of willingness to smile and confusion [23,24,25]. 

As far as we are concerned a limitation of our research was usage of the questionnaire. It might happened that participants’ were trying to guess ‘the proper’ answer and were not honest while filling it in. Another limitation was the number of the participants- the control group was smaller than the treatment one. At the very beginning of our research we included the same number of participants to both groups (60). However, not all the patients appeared at the second dental appointment-3 from the treatment and 19 from the control one resigned. In our opinion the fact that more people from the control one resigned may be associated with the fact that diabetic patients paid more attention to obtained information on relationship of oral hygiene with diabetes treatment and were more focused on marinating the optimal health.

## 5. Conclusions

Professional oral hygiene training improves oral status of diabetic patients. There is a need of dental practitioners being included in a multidisciplinary teams treating diabetic patients.

## Figures and Tables

**Table 1:** Statistically significant results for difference in means between Control and Treatment groups (*n* = 88).

Factor	Test	Visit 1		Test Statistics	*p*	${\hat{r}}_{\mathrm{biserial}}^{\mathrm{rank}}$
		Control Group (SD)	Treatment Group (SD)			
Oral Hygiene Index (DI)	*np*	1.60 (0.70)	2.00 (0.70)	573.00	0.006	−0.35
Oral Hygiene Index (CI)	*np*	1.10 (0.65)	1.50 (0.70)	594.00	0.011	−0.33
Oral Hygiene Index (CI + DI)	*p*	2.88 (0.93)	3.43 (0.96)	−2.61	0.011	−0.57 *
modified Sulcus Bleeding Index	*np*	0.17 (0.16)	0.52 (0.37)	277.50	<0.001	−0.69

Annotation: *np*–non-parametric; *p*–parametric; * effect size Hedges *g*.

**Table 2:** Estimating the differences in means for two independent samples for OHI-S Index and its components.

Factor	Test	Visit 2		Test Statistics	*p*	${\hat{r}}_{\mathrm{biserial}}^{\mathrm{rank}}$
		Control Group (SD)	Treatment Group (SD)			
Oral Hygiene Index (DI)	*np*	1.50 (0.85)	1.00 (0.70)	1400.00	<0.001	0.58
Oral Hygiene Index (CI)	*np*	1.30 (0.80)	1.80 (0.40)	534.00	0.002	−0.40
Oral Hygiene Index (CI + DI)	*p*	2.83 (0.92)	2.66 (0.66)	0.89	0.379	0.20 *

Annotation: *np*–non-parametric; *p*–parametric; * effect size Hedges *g*.

**Table 3:** Estimating the significance of differences in means for two dependent samples (*n* = 57).

Factor	Test	Treatment Group		Test Statistics	*p*	${\hat{r}}_{\mathrm{biserial}}^{\mathrm{rank}}$
		Visit 1 (SD)	Visit 2 (SD)			
Approximal Plaque Index	*np*	75.00 (27.80)	68.20 (25.00)	1200.00	<0.001	0.82
Oral Hygiene Index (DI)	*np*	2.00 (0.70)	1.00 (0.7)	1600.00	<0.001	1.00
Oral Hygiene Index (CI)	*np*	1.50 (0.70)	1.80 (0.40)	445.50	0.017	−0.38
Oral Hygiene Index (CI + DI)	*p*	3.43 (0.95)	2.66 (0.66)	8.58	<0.001	1.12
Modified Sulcus Bleeding Index	*np*	0.52 (0.37)	0.42 (0.28)	120.00	<0.001	0.51

Annotation: *np*–non-parametric; *p*–parametric.

## Data Availability

The data presented in this study are available on request from the corresponding author.
